# Prevalence of Carpal Tunnel Release as a Risk Factor of Trigger Finger

**DOI:** 10.29252/wjps.9.2.174

**Published:** 2020-05

**Authors:** Yousef Shafaee-Khanghah, Hossein Akbari, Nima Bagheri

**Affiliations:** Department of Plastic and Reconstructive Surgery, Hand and Microsurgery, Hazrat-E-Fatemeh Hospital, Iran University of Medical Sciences, Tehran, Iran

**Keywords:** Carpal tunnel syndrome, Trigger finger, Prevalence

## Abstract

**BACKGROUND:**

Carpal tunnel release (CTR) is acknowledged as a predisposing factor for the development of the trigger finger. However, the incidence of new-onset trigger finger after CTR surgery has been inconsistently reported. In this study, we aimed to evaluate the prevalence of CTR as a risk factor of the development of the trigger finger.

**METHODS:**

In a retrospective study, 57 consecutive patients who underwent surgery for the treatment of trigger finger were included. The severity of carpal tunnel syndrome (CTS) was determined using the electromyogram test and nerve conduction study. The clinical and demographic characteristics of the patients were extracted from their medical profiles and compared between patients who did and did not develop a trigger finger after CTR.

**RESULTS:**

Post-CTR trigger finger was detected in 15 (26.3%) patients. The trigger finger occurred approximately six months after CTR surgery. The thumb and ring fingers were the most commonly involved fingers. Ten out of 15 (66.7%) patients who developed a post-CTR trigger finger had mild-to-moderate CTS, and five (33.3%) patients had severe CTS. No significant difference was found between the patients who did and did not develop a trigger finger after CTR.

**CONCLUSION:**

The rate of developing a post-CTR trigger finger was remarkable in our study. Therefore, the potential sequelae should be discussed with patients, preoperatively.

## INTRODUCTION

A mismatch between the volume of the flexor tendon sheath and flexor tendon causes a situation named the trigger finger or stenosing tenosynovitis. This mismatch generally occurs at A-1 pulley and prevents the smooth gliding of the flexor tendon at flexion and extension.^1^ Trigger finger is one of the most common causes of pain and disability in the adult population, with a prevalence of roughly 2% in the general population.^2^ Treatment modalities include splinting, corticosteroid injection, and surgical release, which are tailored to the severity of symptoms.^3^

Although repetitive finger movements and trauma have been proposed as the underlying etiology of trigger finger,^3^^,^^4^ the exact etiology of this disorder is not elucidated. However, patients with certain conditions such as diabetes, rheumatoid arthritis, and obesity are at higher risk of developing a trigger finger.^1^

Carpal tunnel syndrome (CTS) is the most frequent type of peripheral nerve entrapment syndrome. It is the result of the median nerve compression at the wrist, caused by the increased pressure in the carpal tunnel. The main symptoms of CTS include numbness, weakness, and pain.^5^ A prevalence of 3.8% has been reported for CTS in the general population.^6^ Decompression of the median nerve named as carpal tunnel release (CTR) is the surgical treatment of CTS.^7^ The exact etiology of CTS is unknown. However, similar to trigger finger, certain conditions such as diabetes, arthritis, and obesity are commonly associated with carpal tunnel syndrome.^8^

The tendency of CTS and trigger finger to coexist in the same patient suggested the possibility of a common pathophysiological process.^9^ Later investigations revealed that CTR surgery might predispose the development of a trigger finger. The study of Lee *et al.* revealed that patients with greater volar migration of the flexor tendons after CTR are more likely to present with post-CTR trigger finger.^10^ Despite this evidence, the development of trigger finger after CTR has been inconsistently reported, and therefore, the clinical scenario of patients presenting with trigger finger following the CTR is controversial.^11^^,^^12^ In this study, we aimed to analyze the prevalence of CTR as a risk factor of trigger finger in a single-center retrospective study to shed more light on this controversial subject.

## MATERIALS AND METHODS

This research was approved by the review board of our institute under the code of IR.IUMS.REC.1398.927, and patients provided the written consent to use their medical data. The study protocol complies with the Declaration of Helsinki. In a retrospective study, patients who underwent surgical treatment for trigger finger at our center between 2014 and 2017 were included. Patients whose medical records were not available were excluded from the study. From a total of 72 patients, 57 patients had complete medical data and were analyzed in this study. The surgeries were performed by four different hand surgeons and with the same method.

The CTS severity was classified using the electromyogram (EMG) test and nerve conduction study (NCS). Accordingly, the CTS was categorized into mild, moderate, and severe types. Mild CTS was defined as slow orthodromic sensory conduction velocity (<44 m/s) and normal motor distal latency. Moderate CTS was defined as slow orthodromic sensory conduction velocity (<44 m/s) and motor distal latency prolonged by more than 4.4 m/s. Severe CTS was defined as prolonged or absent motor distal latency as well as an absent sensory nerve action potential. The clinical and demographic characteristics of the patients were extracted from the patients’ profiles and included age, gender, body mass index (BMI), the involved finger, history of associated diseases such as diabetes, rheumatoid arthritis, hypothyroidism, etc. 

Statistical analyses were performed by SPSS software for Windows (Version 16, Chicago, Illinois, USA). Descriptive data were provided as mean±standard deviation or number and percentage. Comparison of the mean values between the two study groups was made using an independent t-test or its nonparametric counterpart (Mann-Whitney U test). Comparison of qualitative variables was made using a Chi-Squared test. A p value of less than 0.05 was considered significant.

## RESULTS

A total of 57 patients who underwent surgical treatment for trigger finger were included in this study. The study population consisted 13 (22.8%) males and 44 females (77.2%) with the mean age of 60.2±14.3, ranging from 29 to 78 years. The mean BMI of the patients was 30.4±5.3 kg/m^2^, ranging from 23.1 to 33.4 kg/m^2^. Diabetes, rheumatoid arthritis, and hypothyroidism were detected in 5 (8.7%), 1 (1.7%), and 1 patient (1.7%), respectively. In 23 (40.4%) patients, trigger finger occurred in the thumb. In 28 (49.1%) patients, trigger finger was noticed in the ring finger. In 6 (10.5%) patients, trigger finger happened in the long finger.

Out of 57 patients who underwent surgery for trigger finger, 15 (26.3%) patients had a history of CTR, including 5 (33.3%) males and 10 (66.7%) females. The CTS was mild-to-moderate in 10 (66.7%) patients and severe in 5 (33.3%) subjects. The severity of CTS was not significantly different between the male and female populations (*p*=0.56). In 4 (26.7%) patients, trigger finger was noted eight months after the CTS surgery. In 5 (33.3%) patients, trigger finger was observed seven months after the CTS surgery. In 4 (26.7%) patients, trigger finger was seen six months after the CTS surgery. In 2 (13.3%) patients, trigger finger was visible five months after the CTS surgery. The mean duration between CTR and onset of trigger finger was 6.7±1.6 months. Three patients had a history of simultaneous CTR and trigger finger surgery in the thumb. These patients developed a trigger finger later in the ring finger of the same hand. 

The mean age of the patients was 61.5±14.5 years in patients with a history of CTR and 59.8±18.3 years in patients without a history of CTR (*p*=0.86). The mean BMI of the patients was 31.2±5.2 kg/m^2^ in patients with a CTR history and 30.1±5.4 kg/m^2 ^in patients without a CTR history (*p*=0.77). The thumb, ring, and long finger were involved in 4 (26.8%), 10 (66.6%), and 1 (6.6%) patients with a CTR history and 19 (45.2%), 18 (42.8%), and 5 (12%) patients without a CTR history (*p*=0.28). Also, the distribution of associated disease was not significantly different between patients with and without a CTR history (*p*=0.53). The clinical and demographic characteristics of the patients with and without a history of CTS have been compared in Table 1.

**Table 1:** Comparison of demographic and clinical characteristics between the patients with and without a history of carpal tunnel release

**Variable**	**CTR patients** **(n=15)**	**Non-CTR patients** **(n=42)**	*p* ** value**
Age (year)	61.5±14.5	59.8±18.3	0.86
Gender			
• Male • Female	5 (33.3%) 10 (66.7%)	8 (19%) 34 (81%)	0.6
BMI (kg/m^2^)	31.2±5.2	30.1±5.4	0.77
Involved finger			
• Thumb • Ring • Long	4 (26.8%) 10 (66.6%) 1 (16.6%)	19 (45.2%) 18 (42.8%) 5 (12%)	0.28
Associated disease			
• Diabetes • Rheumatoid arthritis • Hypothyroidism	2 (13.3%) - -	3 (7.2%) 1 (2.3%) 1 (2.3%)	0.53

CTR: carpal tunnel release; BMI: body mass index. Data were presented as mean±SD or number (%). *p*<0.05 was considered significant

## DISCUSSION

In this research, we evaluated the prevalence of CTR as a predisposing factor for the trigger finger. Based on the results of this study, 15 out of 57 patients who underwent surgical treatment for trigger finger had a history of CTR. Therefore, a prevalence of 26.3% was obtained for CTR as a predisposing factor of trigger finger. No significant difference was found ‏between the clinical and demographic characteristics of the patients with and without a history ‏of CTR.‏

The incidence of trigger finger following the CTR has been inconsistently reported. Lin *et al.* ‏conducted a retrospective cohort study using the longitudinal health insurance database 2000, from the National Health Insurance in Taiwan. They identified 2605 CTS patients who received CTR. For each CTR patient, four age and sex-matched CTS patients without a CTR history were included as the control cohort (non-CTR group, n=10,420). The overall risk of trigger finger in the CTR group was 3.63-fold greater than the non-CTR group. The incidence of post-CTR trigger finger was the highest in the first six months and significantly decreased afterward. Diabetes, wrist fracture, and end-stage renal disease were significantly more prevalent in the CTR group than in the non-CTR group.^12^

Similar to the study of Lin *et al.*, the mean duration from the CTR to the onset of the trigger finger was approximately 6 months in the present series. However, while Lin *et al.* found several associations between the post-CTR trigger finger and comorbidities, we detected no such correlations. This inconsistency could be attributed to the smaller patients’ numbers in the present series. El-Hadidi evaluated the association between the CTR and trigger finger in 565 consecutive cases, who were operated by the open release of transverse carpal ligament. Triggering after open CTR occurred in 59 patients (10.44%). Comorbid diabetes mellitus was detected in 11 out of these 59 patients (18.6%).^13^

Triggering after open CTR was detected in 26.3% of the patients in the present series, which was considerably more than the study of El-Hadidi.^13^ Comorbid diabetes was identified in 13.3‎‏% of ‏patients with triggering after CTR, which was comparable with the results of the study of ‏‎ El-‎Hadidi.^13^^‎^ Goshtasby *et al.* aimed to identify the risk factors for the new-onset of trigger finger after CTR. In a retrospective chart, they reviewed multiple variables in 792 CTR patients. They also compared the variables between patients who did and did not develop trigger finger after CTR. The incidence of trigger finger after CTR was 6.3% in this study. In multivariate analysis, osteoarthritis and undergoing an endoscopic procedure were identified as the only independent risk factors.^14^

The incidence of new-onset triggering was remarkably more in the present series (26.3% vs. 6.3%). However, we did not evaluate the role of osteoarthritis and endoscopic procedure as the risk factors of developing a trigger finger following the CTR. Zhang *et al.* aimed to elucidate the association between CTR and the subsequent development of the trigger finger. In a retrospective study, 1,386 hands from 1,140 patients who underwent primary CTR were evaluated. A new-onset trigger finger was detected in 147 (10.6%) patients within one year before CTR and in 81 (5.8%) patients within one year after CTR. Increased BMI was statistically associated with the development of trigger finger after CTR.^15^

The development of new-onset trigger finger was significantly more in the present study (26.3% vs. 5.8%). Moreover, we did not find any significant association between the BMI and development of post-CTR trigger finger. The study of Hayashi *et al.* revealed that CTR surgery is a significant risk factor for the development of trigger finger and may accelerate the onset of trigger finger, when CTS was mild to moderate, but not in severe CTS.^16^ In the present series, 10 out of 15 patients who developed a post-CTR trigger finger had mild-to-moderate CTS, and 5 patients suffered from severe CTS.

Lin *et al.* aimed to assess the prevalence of trigger finger development after CTR surgery in a systematic review. From a total of 5654 CTR surgeries that were evaluated in this series, 483 patients (8.5%) developed trigger finger after CTR. The reported incidence of new-onset trigger finger after CTR surgery ranged from 5.2% to 31.7%. The time to development of post-CTR trigger finger was approximately six months. The thumb and ring finger were the most commonly involved triggered fingers.^11^

The results of the present study were consistent with the results of this review in several aspects. First, the incidence of post-CTR trigger finger in our series was in the range reported in this review. Second, the development of the trigger finger was approximately six months after CTR surgery. Third, the thumb and ring finger was the most commonly involved triggered finger. The present study was not without limitations. The main limitation of this study was the small number of patients, particularly in the post-CTR trigger finger group. This limitation might have adversely affected the power of statistical analysis. Besides, the small number of patients did not allow the multivariate analysis of the data. CTR could be regarded as a predisposing factor for the development of a trigger finger. In this study, the prevalence of CTR in trigger finger patients was 26.3%. This rate of prevalence is not negligible, and therefore, the risk of developing trigger finger should be discussed with patients preoperatively, as potential sequelae.
